# Identity and pathogenicity of some fungi associated with hazelnut (*Corylus avellana* L.) trunk cankers in Oregon

**DOI:** 10.1371/journal.pone.0223500

**Published:** 2019-10-10

**Authors:** Nik G. Wiman, John Bryan Webber, Michele Wiseman, Lea Merlet

**Affiliations:** 1 Oregon State University, Department of Horticulture, North Willamette Research and Extension Center, Aurora, Oregon, United States of America; 2 Oregon State University, Department of Horticulture, Corvallis, Oregon, United States of America; 3 Oregon State University, Department of Botany and Plant Pathology, Corvallis, Oregon, United States of America; Universita del Salento, ITALY

## Abstract

Four fungi isolated from trunks and branches of European hazelnut (*Corylus avellana* L.) from commercial orchards in the Willamette Valley, Oregon were characterized and pathogenicity was tested on potted hazelnut trees. The acreage of hazelnuts in Oregon has expanded greatly in recent years in response to the availability of Eastern filbert blight resistant cultivars. Fungi were characterized using the BLASTn algorithm and the GenBank database with multiple partial gene sequence(s). If BLASTn and GenBank were not sufficient for species-level identification, then a multilocus sequence analysis (MLSA) was performed. The four pathogens were identified as *Diplodia mutilla* (Fr.) Mont., *Dothiorella omnivora* B.T. Linaldeddu, A. Deidda & B. Scanu, *Valsa* cf. *eucalypti* Cooke & Harkn., and *Diaporthe eres* Nitschke. All pathogens but *D*. *omnivora* have not been previously reported from European hazelnut in the literature. All four pathogens caused lesions on trunks bare root hazelnut trees cv. ‘Jefferson’ planted in pots in the greenhouse and fungi were re-isolated from inoculated trees. *D*. *mutilla* appeared particularly aggressive in repeated inoculation experiments.

## Introduction

The US production of European hazelnut (*Corylus avellana* L.) occurs almost exclusively (>98%) in Oregon’s Willamette Valley, which has an ideal climate and suitable soils for production. Until recently, the hazelnut industry in the Pacific Northwest, which once represented substantial acreage in western Washington and British Columbia, had been in a decades-long decline because of the devastating effects of the adventive fungal pathogen *Anisogramma anomola* (Peck). *A*. *anomola* is endemic to *C*. *americana* Walt., one of three native North American hazelnut species that is common across the Midwestern US and East Coast. On the native host, *A*. *anomola* is a minor pathogen, but on European hazelnut it causes the devastating disease known as Eastern filbert blight (EFB). Symptoms of the disease include branch dieback and perennial cankers that can ultimately kill the tree if they are not removed [1–3]. Pruning of cankers during winter and spraying of fungicides from bud break through shoot elongation have become standard management tactics for EFB in susceptible hazelnut cultivars, and loss of mature acreage has been somewhat curbed by the success of the EFB management program [4].

While EFB can be successfully managed with fungicides in susceptible cultivars [5], the best long-term management strategy is to breed cultivars with genetic resistance. Release of European hazelnut cultivars that are resistant to EFB by the Oregon State University hazelnut breeding program [6–8] has stimulated a period of intense growth in the Oregon hazelnut industry, with the planted acres in the Willamette Valley more than doubling in the last 10 years to more than 31,565 ha (78,000 ac) (Pacific Agriculture Survey LLC, personal communication). Most, if not all of the new plantings consist of cultivars with single gene resistance to EFB.

Now that EFB is less of a concern in the new hazelnut cultivars, previously unrecognized disease symptoms may be becoming more apparent. While it is possible that the new cultivars will have different problems, lack of long-term experience with these new cultivars makes that possibility difficult to assess. Hazelnuts can be regarded as a relatively permanent crop that reaches maturity in about 12 years with the potential to remain productive for 40–50 years [9] The first hazelnut cultivar with genetic resistance to EFB was released in 2009 [10], thus, the Oregon hazelnut industry lacks long-term experience with the new cultivars. Recently, field visits to commercial hazelnuts farms growing EFB-resistant cultivars to investigate reports of decline symptoms have resulted in isolation of previously unassociated fungal pathogens from trunks and branch cankers. Observed symptoms vary, but they include dieback of branches, low nut production, small or poorly filled nuts, slow bud break, stunted leaves, poor growth and presence of cankers on main trunks or branches that are not associated with *A*. *anomala*.

There may be several factors contributing to the emergence of previously unknown trunk diseases in hazelnuts. One explanation is increased incidence of canker diseases in response to climate change and its effects on plants and pathogens [11,12]. Another potentially important factor is reduced fungicide use in the new orchards. In orchards with legacy EFB-susceptible cultivars, four fungicide cover sprays are applied every two weeks from bud swell to early shoot elongation; in the new EFB-resistant plantings, spring applied fungicides are recommended only for the first season after planting if there are diseased legacy orchards nearby [5,13]. Notably, many orchards with EFB-resistant cultivars receive no fungicides at all except for a fall application of copper against *Xanthomonas arboricola* pv. *corylina*, the causal agent of bacterial blight of hazelnut [13]. Management practices may also cause wounds or plant stress that can be exploited by pathogens. Hazelnut production has largely expanded on suboptimal planting sites with heavy soils, where trees may be more susceptible to disease because of stressful growing conditions. Intense pruning practices and herbicide use for management of basal adventitious shoots (suckers and watersprouts) may also contribute to the problem by providing entry pathways for pathogens [14]. Finally, with greater genetic diversity of hazelnut cultivars and rapidly expanding acreage of hazelnut in the Willamette Valley, there may be more opportunity for new diseases to establish.

Pathogenicity was examined for four fungi isolated from symptomatic European hazelnut trees collected from commercial orchards in the Willamette Valley, Oregon. To characterize the fungi, we used the BLASTn algorithm and the GenBank database with multiple partial gene sequence(s). If BLASTn and GenBank were not sufficient for species-level identification, then a multilocus sequence analysis (MLSA) was performed. Three of the pathogens have not been previously reported from European hazelnut in the literature. The pathogenicity of these four fungi was evaluated by inoculating potted bare root EFB-resistant hazelnut trees under greenhouse growing conditions.

## Materials and methods

### Isolation of fungi

Materials were collected from agricultural settings with permission of landowners. Symptomatic plant material showing stem and trunk cankers was collected from commercial hazelnut orchards in the Willamette Valley, OR during the 2016 and 2017 growing seasons and brought to the Oregon State University Plant Disease Clinic in Corvallis, OR. Stem cankers were surface disinfested in 10% bleach for 3 min, rinsed for 1 min in deionized water, and allowed to dry in a laminar flow hood. Bark tissue was aseptically shaved off and then cambial tissues from the canker margins were excised and transferred to streptomycin amended potato dextrose agar (SPDA) and water agar (WA). SPDA and WA plates were incubated in total darkness at 20°C. There were four fungal isolates that we selected for molecular identification and inoculation experiments. These were assigned plant clinic numbers 17-288A, 17-288C, 17-228B, and 16-1224A.

### Molecular identification of fungi

Total genomic DNA was extracted from 7-day-old hyphal-tipped axenic cultures, grown on SPDA at ambient temperature, using the Fast DNA^®^ SPIN Kit and the FastPrep^®^ Instrument (MP Biomedicals, Santa Ana, CA). Partial gene sequence of the nuclear ribosomal internal transcribed spacer region (ITS rDNA, including ITS1, 5.8S, and ITS2 regions) was amplified using the primers ITS1 (5’-TCCGTAGGTGAACCTGCGG-3’) with ITS4 (5’-TCCTCCGCTTATTGATATGC-3’) (White et al., 1990), translation elongation factor 1-alpha (*tef1*) using EF1-728F (5’-CATCGAGAAGTTCGAGAAGG-3’) with EF1-986R (5’-TACTTGAAGGAACCCTTACC-3’) (Carbone & Kohn, 1999), and β-tubulin gene (*tub2*) using the primers Bt-2a (5’-GGTAACCAAATCGGTGCTGCTTTC-3’) and Bt-2b (5’-ACCCTCAGTGTAGTGACCCTTGGC-3’) [15]. The PCR reaction mixture consisted of 0.2 μM of each primer, 1x AccuStart II PCR ToughMix (Quantabio, Beverly, MA, USA), 1x gel loading dye, and 0.5–50 ng of template DNA in a final volume of 25 μL. Amplifications were conducted in a C1000 Touch^™^ Thermal Cycler (Bio-Rad, Hercules, CA, USA).

For ITS and *tef* products, amplification started with an initial denaturation cycle of 3 min at 95°C, followed by 35 cycles of 30s at 95°C, 45s at 57°C, and 60s 72°C and a final extension cycle of 5 min at 72°C. For *tub2* products, amplification started with an initial denaturation cycle of 3 min at 95°C, followed by 35 cycles of 30s at 95°C, 30s at 52°C, and 30s 72°C and a final extension cycle of 5 min at 72°C. Successful amplification was verified on a 1.5% agarose gel post-stained with ethidium bromide. PCR amplicons were then treated with ExoSAP-IT^™^ as directed by the manufacturer (Affymetrix Inc., Santa Clara, CA, USA) and sequenced with forward and reverse primers using a ABI 3730 capillary sequence machine by the Center for Genome Research and Biocomputing (OSU, Corvallis, OR, USA). Contigs were hand-edited and assembled de novo using Geneious 9 (Biomatters Ltd., Auckland, NZ).

Assemblies were compared to type accessions in the GenBank database using the BLASTn algorithm. Multilocus sequence analyses were executed when type accessions were available for multiple loci. The best-fit model of sequence evolution for the MLSA datasets were selected and model parameter estimates obtained using jModeltest 2.1.3 [16]. The Bayesian analyses were performed using MrBayes v. 3.2.6 [17] with the Markov Chain Monte Carlo (MCMC) algorithm, 10% burn-in, 4 heated chains, and a sub-sampling frequency of every 1,000 Markov chains. The MCMC analysis ran until the average standard deviation of split frequencies fell below 0.01.

### Inoculations

There were three inoculation experiments conducted with the fungal isolates. The first two evaluated 16-1224A (*Diplodia mutila* (Fr.) Mont.) alone, which appeared to be the most aggressive of the fungal isolates. The third inoculation experiment included all four isolates.

For the first inoculation experiment, trunks of potted 3-year-old hazelnut trees (c.v. ‘Jefferson’) were surface disinfested with 70% ethanol, rinsed with de-ionized water, dried, and then inoculated with 4 mm plugs of 4-day-old *D*. *mutila* grown on SPDA. Each of 5 trees were inoculated twice, once each on opposite sides of them stem, 76 mm (3 in) above the soil line using a sterile 4 mm cork borer. For each of these trees 4-day-old *D*. *mutila* plugs were placed on the 3–4 mm deep wounds and then the inoculation site was wrapped in Parafilm. A 6^th^ tree was inoculated with SPDA blank plugs as a control. Inoculated hazelnuts were incubated under greenhouse conditions (17 to 26°C with temperature-controlled venting and no supplemental lighting).

In a second inoculation experiment, potted 1-year-old bare root hazelnut trees (c.v. ‘Jefferson’) were grown out for inoculation with *D*. *mutila* and SPDA blank plugs as described above, except that there was a single inoculation point between two nodes on the stem about 250 mm (10 inches) above the soil level. The total population consisted of 80 potted trees that were randomly assigned to 5 crates each holding 16 trees. Each crate contained 4 trees inoculated with blank SPDA plugs and 12 trees inoculated with plugs of *D*. *mutila*. We destructively evaluated one crate every 14 days post-inoculation (dpi) until no further trees were left to evaluate after 70 days. During evaluations we noted the length of lesions originating from the inoculation site. The randomized block design was analyzed by two-way ANOVA.

For the third inoculation experiment, *D*. *mutila* was inoculated along with three other pathogens, 17-288A (*Dothiorella omnivore* B.T. Linaldeddu, A. Deidda & B. Scanu), 17-288C (*Valsa* cf. *eucalypti* Cooke & Harkn.), and 17-228B (*Diaporthe eres* Nitschke) and blank SPDA plugs as a negative control as described above for potted 1-year-old ‘Jefferson’ trees. There were 16 trees inoculated for each isolate and controls, and 8 trees from each group were evaluated at 28 days (4 weeks) and the other 8 trees were evaluated at 63 days (9 weeks) after treatment. We utilized ANOVA and Tukey HSD to test for differences in canker length between the different isolates at 28 and 63 day dpi. Paired t-tests were used to assess canker growth of individual isolates between the two evaluation periods.

## Results

### Fungal isolation

After 7 days of growth on SPDA media at 25°C, all fungal colonies were initially cottony white in appearance. Isolate #17-228B and #17-288C grew to the edges of the petri dish and developed a pinkish appearance on the underside of the agar plates. Isolate #16-1224a grew vigorously to the edges of the petri dish and developed a deep black coloration on the underside of the agar plates giving the fluffy white mycelium a grayish hue. Isolate #288-A did not reach the edges of the petri dish after 7 days but grew to be matted cottony white and developed the same deep black coloration on the agar plate as #16-1224a. The black coloration of isolates #288-A and #16-1224a continued to darken after 7 days of incubation.

### BLASTn identification

**Fig 1:**
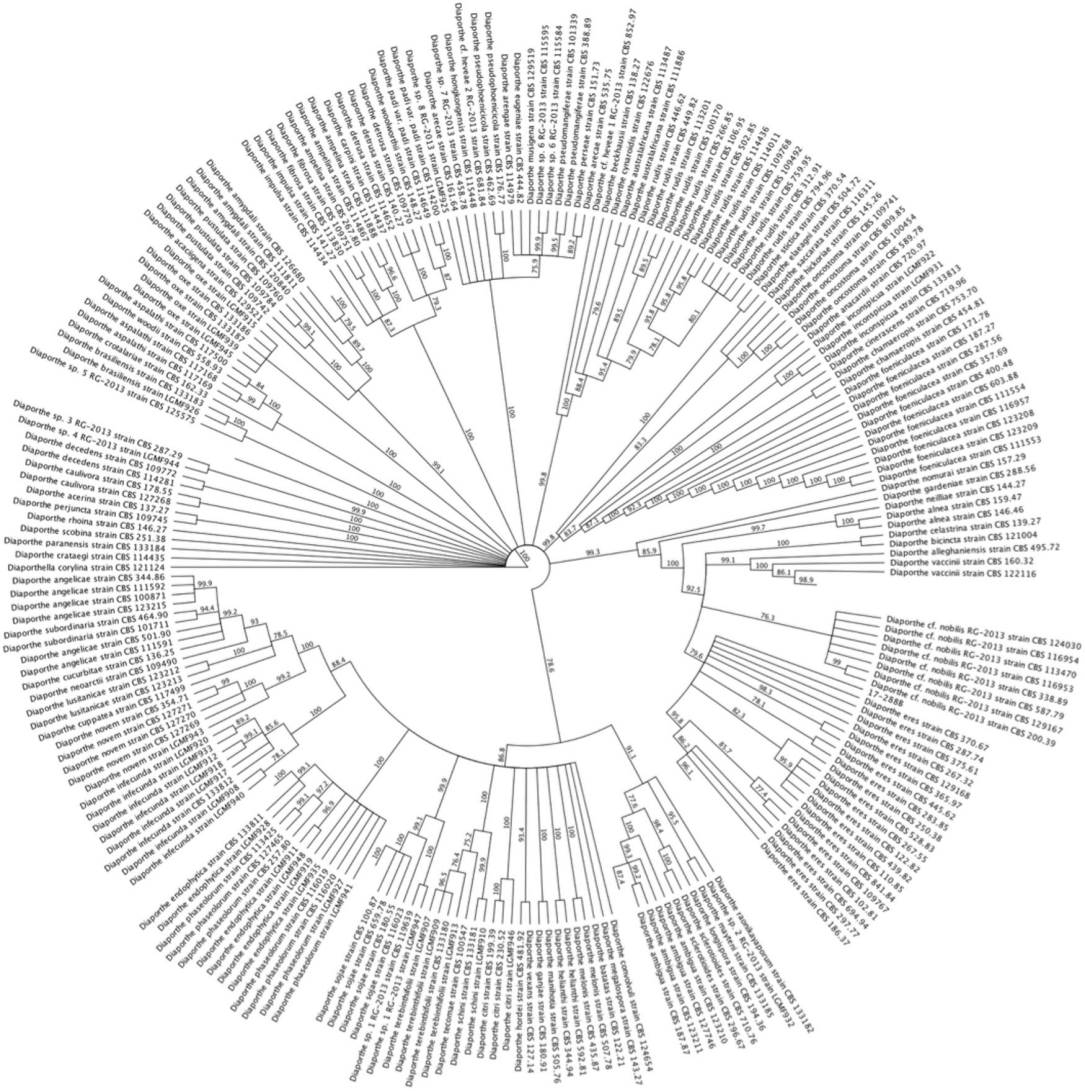
Monophyletic clade of *Diaporthe eres* and the unknown fungus 17-288B.

**Fig 2:**
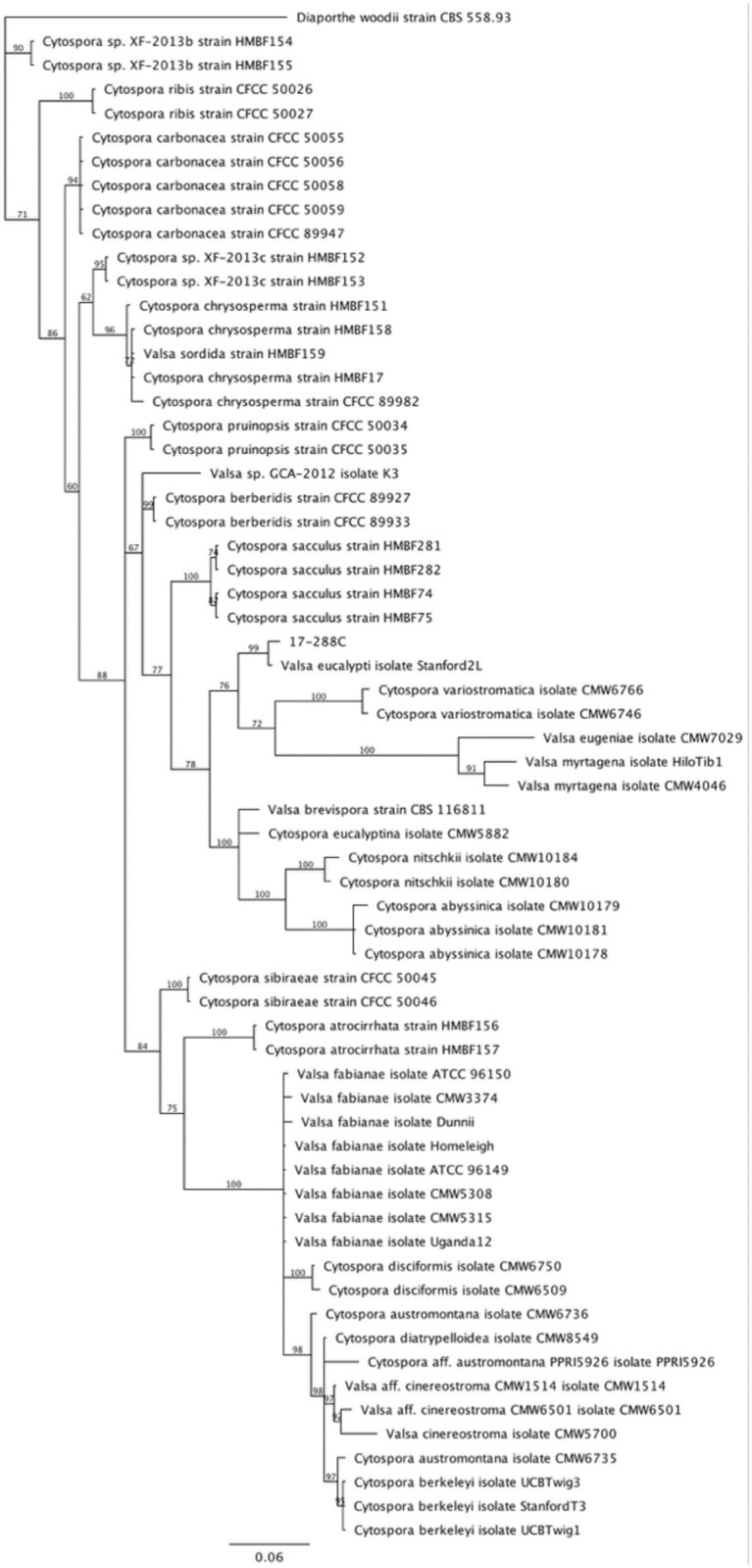
Monophyletic clade of *Valsa eucalypti* and the unknown fungus 17-288C.

Results are summarized in Table 1. Due to insufficient separation, 17-288B and 17-288C were subject to phylogenetic analyses. For the MLST, the best-fit model of nucleotide evolution for Bayesian analyses was determined to by GTR + I + G based on the Bayesian information criterion (BIC). The final dataset had 1626 characters containing concatenated *tef1*, *tub2*, and ITS sequences. The results of the Bayesian analysis revealed a well-supported monophyletic clade of *Diaporthe eres* and the unknown fungus 17-288B (Fig 1). For the ITS analysis the best-fit model of nucleotide evolution for Bayesian analyses was determined to by GTR + I + G based on the BIC. The final dataset had 585 characters containing the nuclear ribosomal internal transcribed spacer region (ITS rDNA, including ITS1, 5.8S, and ITS2 regions). The results of the Bayesian analysis revealed a monophyletic clade of *Valsa eucalypti* and the unknown fungus 17-288C (Fig 2). Since there was very little data from *Cytospora* spp. type specimens in GenBank, the identification should be considered tentative until further sequencing of type specimens has been completed.

**Table 1:** Identification of the four unknown fungal isolates.

Identifier		GenBank Accession Numbers of Top Type Match ^A^		Identification
	ITS	*tef1*	*tub2*	
**16-1224A**	*Diplodia mutila* NR_144906 507/507(100%)	*Diplodia mutila* AY573219 283/283(100%)	*Diplodia mutila* DQ458850 429/429(100%)	*Botryosphaeria stevensii* (*Diplodia mutila*)
**17-288A**	*Dothiorella omnivora* ^B^ KP205497 436/436(100%)	*Dothiorella omnivore* KP205470 247/252(98%)	*Dothiorella sarmentorum* EU673102 381/381(100%)	*Dothiorella omnivora*
**17-288B**	*Diaporthe celastrina* ^B^ NR_152457 549/553(99%)	*Diaporthe ellipicola* KF576245 ^B^ 337/343(98%)	*Diaporthe alleghaniensis* KC843228 498/498(100%)	*Diaporthe eres* ^C^ (*Phomopsis velata***)**
**17-288C**	*Cytospora vinacea* KX256256 386/405(95%)	*Cytospora pruinopsis* KP310849 84/93(90%)	*Digitiseta multidigitate* KY366456 171/181(94%)	*Valsa* cf. *eucalypti* ^D^ (*Cytospora* sp.)

^A^ Number in parenthesis under accession number is the number of nucleotide matches. All accession numbers are from GenBank unless otherwise noted.

^B^ Multiple accessions had identical identity scores; therefore, the best max score accession was chosen.

^C^ Determined by MLSA, see S1 Table.

^D^ Determined by ITS sequence analysis using Adams et al. (2005) *Cytospora* spp. dataset.

### Inoculations

**Fig 3:**
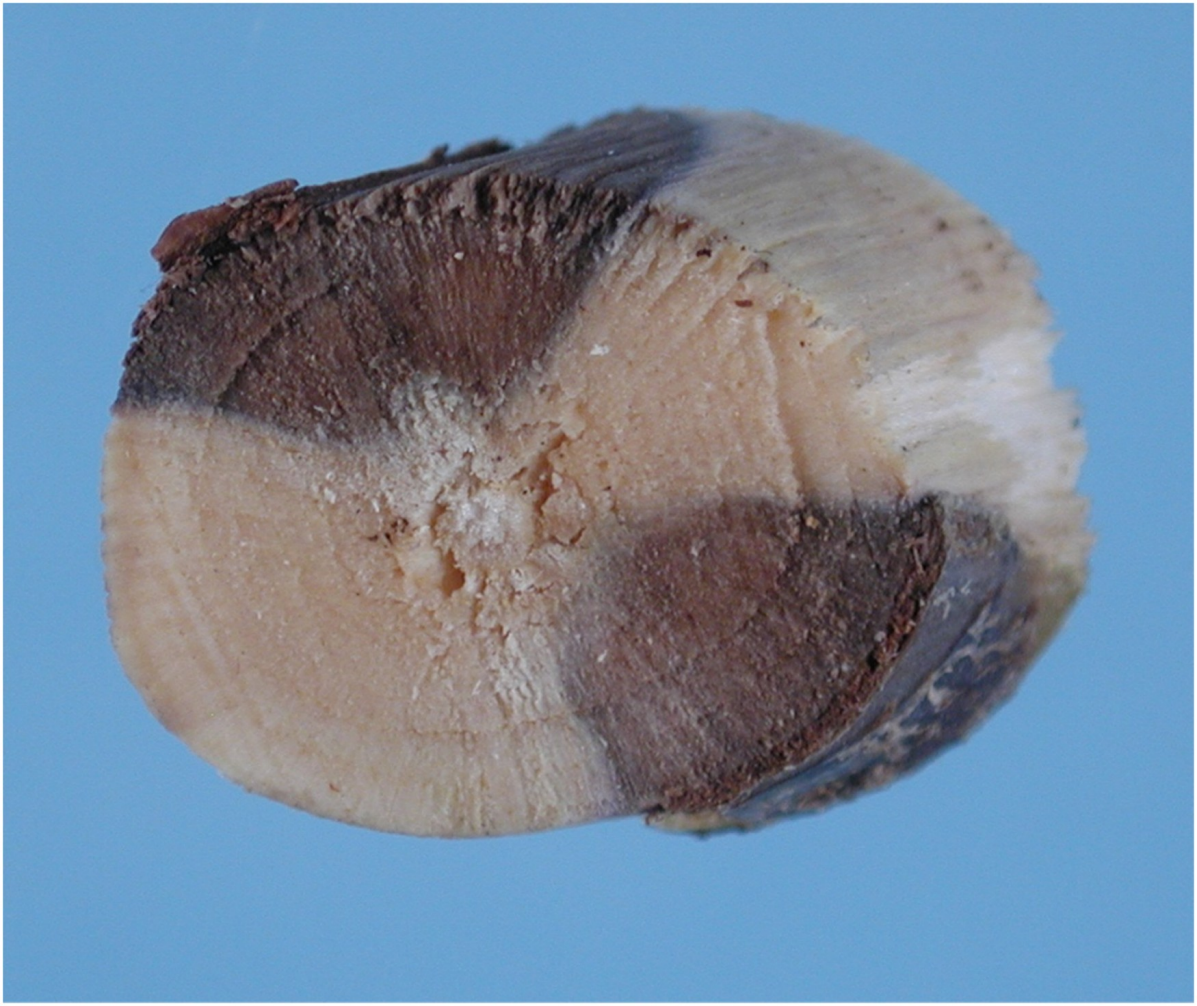
Cross section of a 3-year-old potted hazelnut tree that was inoculated with *Diplodia mutilla* on both sides of the trunk showing lesion growth occluding most of the trunk.

For the initial inoculation experiment on 3-year-old potted hazelnuts, all trees except the negative control developed large lesions that developed above and below the two inoculation sites on both sides of the stem, occluding nearly the entire trunk (Fig 3). Trees inoculated with the fungus exhibited lack of vigor and foliar chlorosis followed by defoliation. The fungus *D*. *mutila* was re-isolated from canker margins on inoculated trees but not from the control tree.

**Fig 4:**
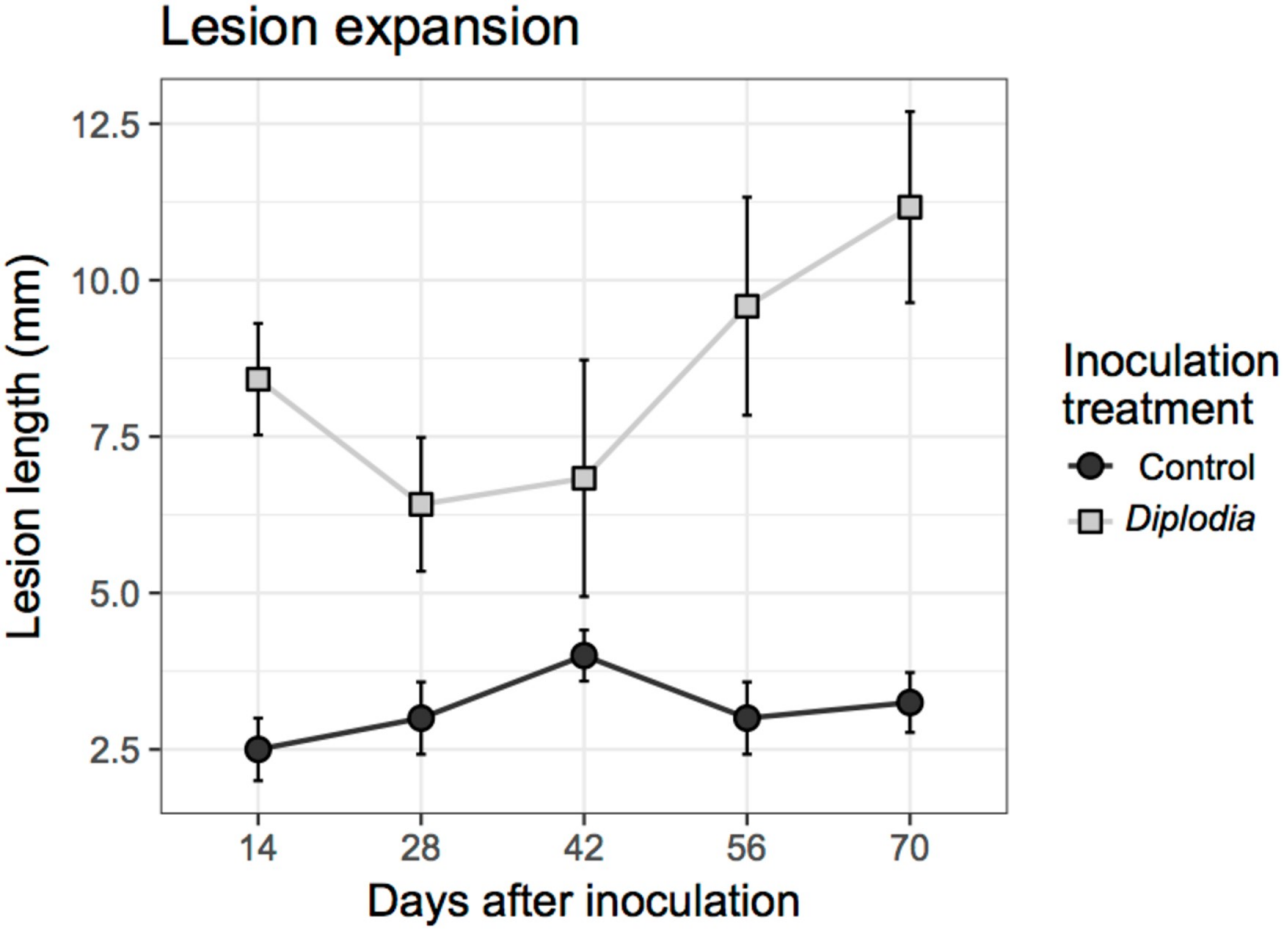
Expansion of lesions caused by *Diplodia mutilla* over time compared to lesions caused by inoculation of sPDA plugs on potted hazelnut trees.

In the second inoculation experiment, *Diplodia mutila* caused vascular discolorations on the 1-year-old hazelnut trunks that were consistently larger and much darker than those caused by inoculation by the SPDA blank plugs (*F*_*1*,*72*_ = 53.50, *P <* 0.0001), and lesions generally increased in size over time (*F*_*3*,*72*_ = 2.76, *P =* 0.0482; Fig 4).

**Fig 5:**
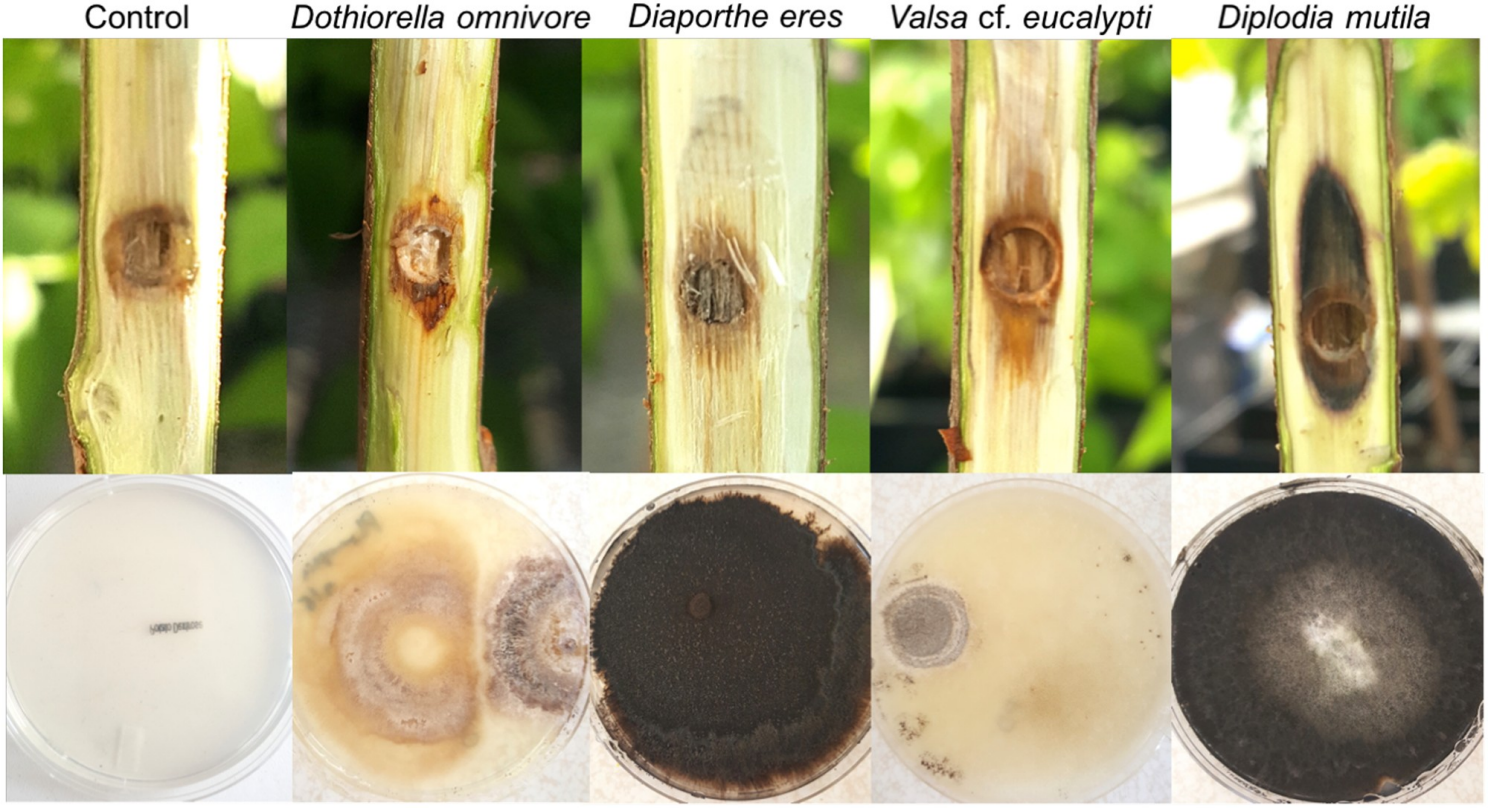
Appearance of the four fungal isolates after four weeks on inoculated trees and growth on sPDA media.

**Fig 6:**
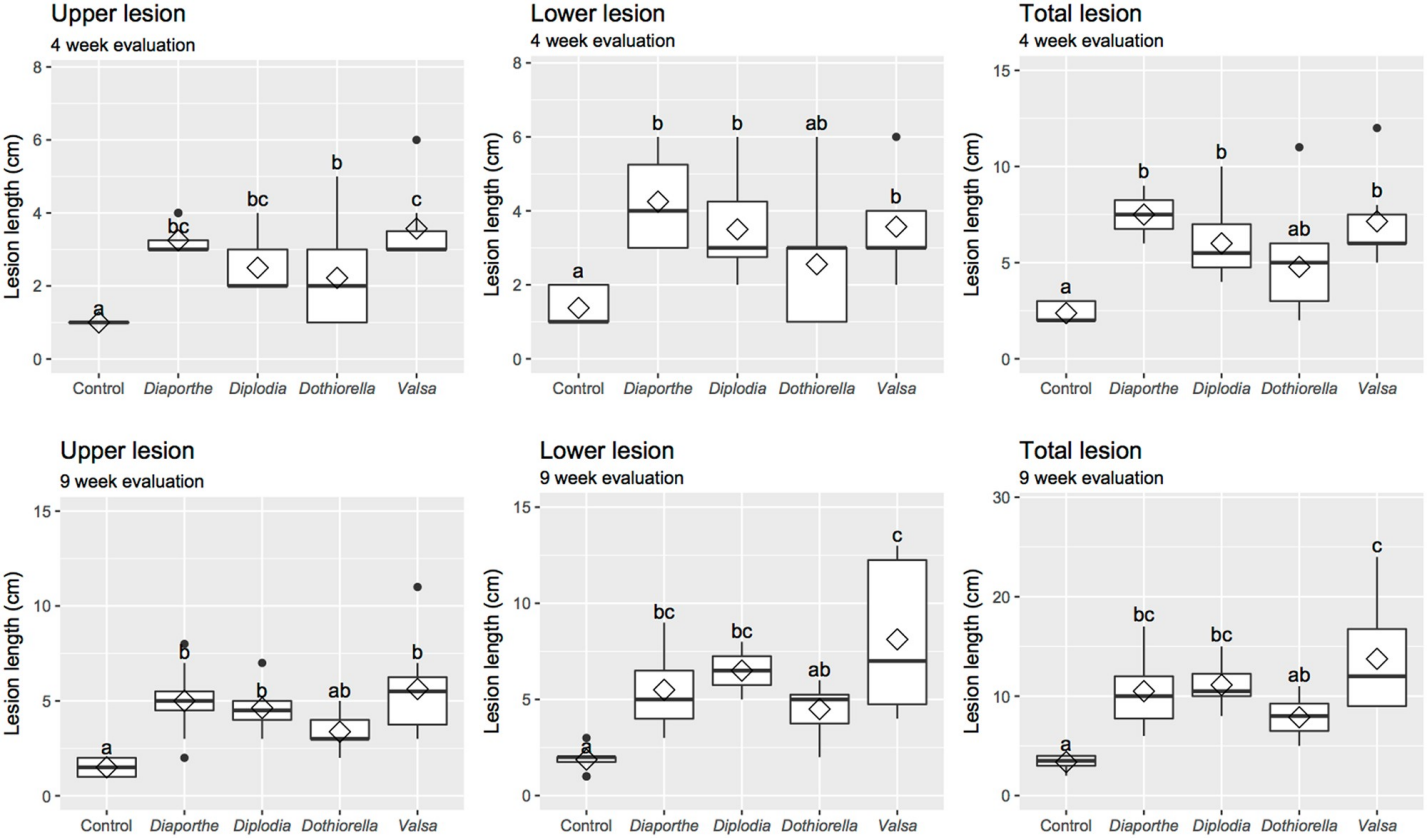
Size of lesions observed in trees inoculated with the four isolates at the 4 and 9 weeks post inoculation (wpi). Diamonds represent treatment means.

In the third inoculation experiment, all fungi that were inoculated caused cankers on the trunks of the potted trees in at least one of the evaluations. Stripping of the bark around the inoculation site revealed lesions that varied in color from brown to black and largely represented the color of the growths on the plates (Fig 5). At 4 weeks post-inoculation (wpi), there were significant differences in the growth of the canker lesions both up (*F*_*4*,*35*_ = 10.20, *P <* 0.0001), and down the trunk from the inoculation site (*F*_*4*,*35*_ = 9.92, *P <* 0.0001), and in the total length of the cankers (*F*_*4*,*35*_ = 8.71, *P <* 0.0001). For most treatments inoculated and evaluated at 4 weeks, there was greater variability in the length of the lower lesion compared to the lesion that moved up the trunk from the inoculation point indicated by greater interquartile range in the boxplots (Fig 6). The length of the upper lesion caused by inoculation by *Dothiorella omnivora* was significantly larger than the upper lesion caused by the control treatment, but the lower lesion and total lesion caused by *D*. *omnivora* was not significantly different from lower or total lesion lengths caused by the control treatment. All other inoculation treatments (*Diplodia mutila*, *Diaporthe eres* and *Valsa* cf. *eucalypti*) caused significantly longer (but equivalent upper, lower, and total lesion length) compared to control treatments at 4 wpi (Tukey HSD; *P <* 0.05; Fig 6).

At 9 wpi, there were significant differences in the growth of canker lesions both up the trunk from the inoculation site (*F*_*4*,*35*_ = 8.51, *P <* 0.0001), down the trunk from the inoculation site (*F*_*4*,*35*_ = 9.11, *P <* 0.0001), and the total length of cankers (*F*_*4*,*35*_ = 11.04, *P <* 0.0001). *Dothiorella omnivora* was the only species did not cause a significantly greater upper, lower and total lesion length compared to controls at 9 weeks. All other fungal inoculation treatments (*Diplodia mutila*, *Diaporthe eres* and *Valsa* cf. *eucalypti*) caused equivalently longer upper, lower, and total canker lesions compared to the control (Tukey HSD; *P <*0.05; Fig 6). Similar to the 4 week evaluations, there was greater variability in the length of the lower lesions compared to the upper lesions indicated by greater interquartile range on the boxplots at the 9 wpi evaluation (Fig 6).

**Fig 7:**
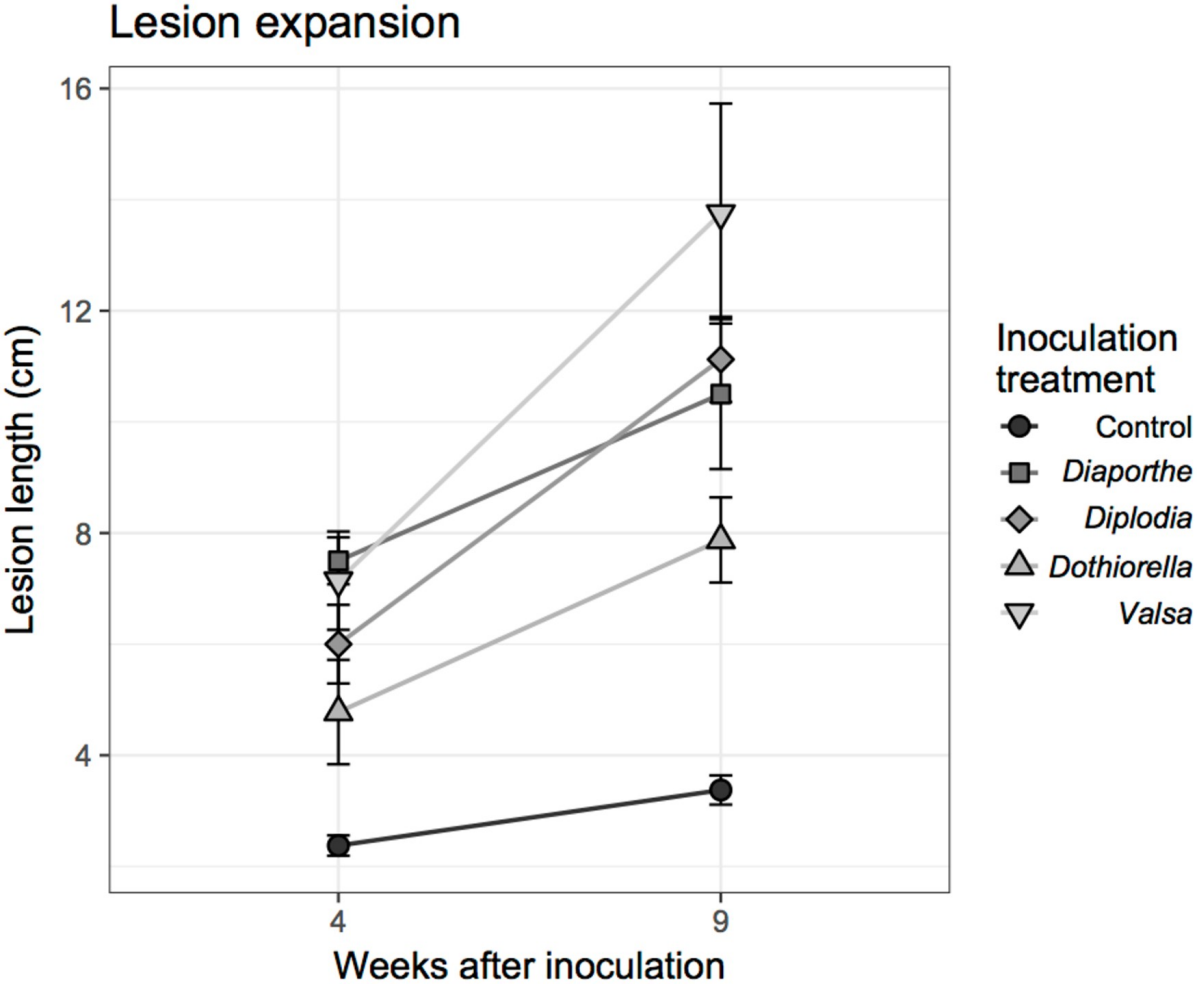
Increase in lesion size for the four isolates between the 4 and 9 weeks post inoculation (wpi).

The smallest mean increase in total lesion length from 4 to 9 weeks was 1 mm for the control treatment (*t =* 3.12, df = 12.49, *P* = 0.004). Over this period, *Diaporthe eres* lesions increased by an average of 3 mm (*t =* 2.12, df = 8.36, *P* = 0.033), *Dothiorella*. *omnivore* lesions increased by 3.10 mm (*t =* 2.55, df = 14.73, *P* = 0.011), *Diplodia mutila* lesions increased by 5.25 mm (*t =* 4.92, df = 13.91, *P* < 0.001), and *Valsa* cf. *eucalypti* lesions increased in length by 6.61 mm (*t =* 3.05, df = 9.63, *P* = 0.006; Fig 7).

All four of the fungal species were successfully re-isolated from the margins of developing canker lesions at each evaluation date. Pathogenicity symptoms were similar to what was first observed in the field and the isolated fungi were verified based on morphological characteristics, thus fulfilling Koch’s postulate. None of these fungal species were re-isolated from the negative SPDA plug control lesions.

## Discussion

We isolated and identified four Ascomycota fungi from hazelnut trees showing symptoms of decline in the Willamette Valley, Oregon and fulfilled Koch’s postulates. These included the *Botryosphaeriaceae* species *Diplodia mutila* and *Dothiorella omnivore*, a species of *Diaporthaceae*, *Diaporthe eres*, and a species of *Valsaceae*, *Valsa* cf. *eucalypti*. Of these species, only *Dothiorella omnivore* has been previously reported as a pathogen of European hazelnut, *C*. *avellana* [18]. The others have not been previously reported as canker pathogens of hazelnut, but are familiar pathogens of perennial tree and vine crops.

Species identification was based on anamorph morphology, culture characteristics, and sequencing data. Teleomorph states were not observed for any of the four fungi. Sequencing multiple regions proved to be sufficient for the identification of both *Diplodia mutila* and *Dothiorella omnivore* while an MLSA was necessary for the determination of *Diaporthe eres*. The identification of *Valsa* cf. *eucalypti* should be considered tentative as there is very little sequence data available for regions other than ITS for the *Valsa/Cytospora* group.

*Botryosphaeriaceae* appear to be an increasing economic problem on a large variety of perennial specialty crops including pistachio, walnuts, grapevines, and almond and other *Prunus* species [19]. For each of these crops, fungal cankers were typically first discovered in isolated instances before spreading and becoming problematic. For example. *Botryosphaeria* was first discovered to cause panicle and shoot blight in California pistachios in an isolated orchard in 1984; by 1998, ideal weather conditions caused the disease to spread and ultimately have a major economic impact on the California pistachio industry [20]. *Botryosphaeriaceae* spp. are generally considered marginally pathogenic endophytic fungi capable of infecting woody hosts in temperate and tropical regions. Symptoms of infection include apical death of twigs and secondary branches and spread through the vascular tissue into flowers and nuts. Laterally cut branch sections show necrotic lesions that travel along the xylem. *Botryosphaeriaceae* can enter the plant host through wounds such as leaf scars, bud scars, peduncle scars, pruning wounds or other mechanical wounds. These fungi are dispersed by two different kinds of spores: 1) pycnidiospores, which are sticky spores spread by water and occasionally insects, and 2) ascospores that spread by wind. Pycnidiospores are most common inoculum form and only need 1.5 hours of free water to germinate [21]. Weakened, diseased, or stressed plants can increase the susceptibility to infection. Disease from *Botryosphaeriaceae* is commonly associated with drought and moisture stress [19]. Inoculum can be spread from old debris, riparian plant species, ornamental plants, other infected crops.

*Diplodia mutila* is an anomorph of *Botyrosphaeria stevensii* Shoemaker, a pathogen that causes branch and trunk cankers on numerous tree species but is well-known from oaks, juniper and ornamental tree species [21–26]. Recently, *D*. *mutila* was reported as a causal agent of branch dieback on walnut in Chile [27], but it has been known from walnut in California for quite some time [28] and has been collected from avocado, citrus, and winegrapes in California [29–31]. *Diplodia coryli* (CBS 242.51) has been reported to cause similar symptoms to *D*. *mutila* on hazelnut in Chile and was previously reported from Europe [32], but was determined to be inauthentic by Phillips et al. 2008 [33], and through phylogenetic analysis, was identified as *Dothiorella vidamera* W.M. Pitt et al [18,34]. Inoculation of *D*. *mutila* consistently caused a significant lesion on the trunks of the hazelnut trees in our studies and it grew aggressively on the plates. It may have grown more aggressively when it was inoculated near the base of the potted tree as in the first inoculation experiment, which may be related to the higher relative humidity in the microclimate at the soil surface.

*Dothiorella omnivora* is a well-known pathogen of wine grape vines [35], and English walnut (*Juglans regia*), but it has also been isolated from *C*. *avellana* in Italy [18]. In both Italy and Oregon hazelnuts and wine grapes are often cultivated in close proximity. Linaldeddu et al. (2016) provided the first description of this pathogen and also tested pathogenicity on *C*. *avellana*. Inoculations produced necrotic lesions around the wound site, but these were no larger than the lesions produced by the control inoculations, suggesting that the pathogen is very weak on hazelnut. However, Koch’s postulates were fulfilled as the fungus was re-isolated from the infected branch and symptoms resembled those first observed when the pathogen was first collected from the field. Our results support the observation that *D*. *omnivore* is a weak pathogen on hazelnut, as this was the only pathogen we tested that did not consistently cause a significantly bigger lesion than the controls. Like Linaldeddu et al. (2016), we successfully re-isolated the fungus from the inoculated trees.

*Diaporthaceae* represent a large group of fungi with excessive species names assigned based on the false assumption of host specificity, as many species are now known to occur on multiple host plants [36]. Species include pathogens, endophytes, saprobes and even mammalian pathogens. In Chile, *D*. *australafricana* is known to cause stem canker and dieback in *C*. *avellana* [37,38]. *Diaporthe eres*, the species that was recovered from symptomatic *C*. *avellana*, is known worldwide as a minor pathogen of woody plants including caneberry [39], peach [40], pear [41], *Vaccinium* spp. [42], and winegrapes [43–45]. However, *Diaporthe eres* has not been previously reported to cause cankers on hazelnut, but *D*. *eres* has been associated with hazelnut kernel mold defects in the Caucasus region [46]

In Europe and in other hazelnut production regions of the world, *Cytospora* canker caused by *C*. *corylicola* is present and has recently become an increasing problem in the important Piedmont hazelnut production region of Italy [47,48]. In our research, we found *Valsa* cf. *eucalypti* causing symptoms very similar to those reported for *C*. *corylicola*. *Cytospora* is the anamorph genus for *Valsa* so this is not surprising. In our trials, *V*. cf. *eucalypti* caused some of the largest lesions at the 9 week evaluation of all the pathogens that we examined.

Considering pathogenicity of these canker-causing fungi, it is important to consider that wounding of the tree for inoculation enhances the ability of the pathogen to penetrate the host. In commercial hazelnut production in Oregon’s Willamette Valley, there are a number of different ways that trunks are inadvertently wounded or stressed during production that could be contributing to increased incidence of these fungi. Trunks are quite frequently damaged by sunburn after plastic trunk guards are removed or canopy management increases sun exposure on trunks. In Oregon hazelnuts are cultivated as single-trunk trees, and management of adventitious shoots (suckers) at the base of the trunk is one of the most challenging production tasks. Trunks are often damaged by repeated application of burn-down herbicides. Failure to control adventitious shoots originating from the trunk in a timely manner sometimes requires a major pruning wound to be made on the trunk. Finally, drought stress is common during summer especially for growers on light soils and limited access to irrigation water. Future studies should examine pathogenicity in the absence of wounding and control strategies. As the Oregon hazelnut industry continues to expand, a formal survey of pathogens associated with canker symptoms is warranted to assess the importance of the different pathogens discussed in this work, and place them in context with other pathogens.

## Supporting information

S1 Table GenBank accessions for taxa used in the *Diaporthe* sp. MLSA. (DOCX)
