# Supplementary material for: Identity and pathogenicity of some fungi associated with hazelnut (Corylus avellana L.) trunk cankers in Oregon
Source: PLoS One. 2019 Oct 10;14(10):e0223500. doi: 10.1371/journal.pone.0223500 (PMC6786572; doi:10.1371/journal.pone.0223500)
Supplement: S1 Table — (DOCX) [file pone.0223500.s001.docx]

| **Taxonomic Identity** | **Strain** | **GenBank Accession Number^1^** | | |
| --- | --- | --- | --- | --- |
|  |  | **ITS** | ***tef1*** | ***tub2*** |
| *Diaporthella corylina* | CBS 121124 | KC343004 | KC343730 | KC343972 |
| *Diaporthe acaciigena* | CBS 129521 | KC343005 | KC343731 | KC343973 |
| *Diaporthe acerina* | CBS 137.27 | KC343006 | KC343732 | KC343974 |
| *Diaporthe alleghaniensis* | CBS 495.72 | KC343007 | KC343733 | KC343975 |
| *Diaporthe alnea* | CBS 146.46 | KC343008 | KC343734 | KC343976 |
| *Diaporthe alnea* | CBS 159.47 | KC343009 | KC343735 | KC343977 |
| *Diaporthe ambigua* | CBS 114015 | KC343010 | KC343736 | KC343978 |
| *Diaporthe ambigua* | CBS 117167 | KC343011 | KC343737 | KC343979 |
| *Diaporthe ambigua* | CBS 123210 | KC343012 | KC343738 | KC343980 |
| *Diaporthe ambigua* | CBS 123211 | KC343013 | KC343739 | KC343981 |
| *Diaporthe ambigua* | CBS 127746 | KC343014 | KC343740 | KC343982 |
| *Diaporthe ambigua* | CBS 187.87 | KC343015 | KC343741 | KC343983 |
| *Diaporthe ampelina* | CBS 111888 | KC343016 | KC343742 | KC343984 |
| *Diaporthe ampelina* | CBS 114867 | KC343017 | KC343743 | KC343985 |
| *Diaporthe ampelina* | CBS 267.8 | KC343018 | KC343744 | KC343986 |
| *Diaporthe amygdali* | CBS 111811 | KC343019 | KC343745 | KC343987 |
| *Diaporthe amygdali* | CBS 115620 | KC343020 | KC343746 | KC343988 |
| *Diaporthe amygdali* | CBS 120840 | KC343021 | KC343747 | KC343989 |
| *Diaporthe amygdali* | CBS 126679 | KC343022 | KC343748 | KC343990 |
| *Diaporthe amygdali* | CBS 126680 | KC343023 | KC343749 | KC343991 |
| *Diaporthe anacardii* | CBS 720.97 | KC343024 | KC343750 | KC343992 |
| *Diaporthe angelicae* | CBS 100871 | KC343025 | KC343751 | KC343993 |
| *Diaporthe angelicae* | CBS 111591 | KC343026 | KC343752 | KC343994 |
| *Diaporthe angelicae* | CBS 111592 | KC343027 | KC343753 | KC343995 |
| *Diaporthe angelicae* | CBS 123215 | KC343028 | KC343754 | KC343996 |
| *Diaporthe angelicae* | CBS 344.86 | KC343029 | KC343755 | KC343997 |
| *Diaporthe angelicae* | CBS 501.9 | KC343030 | KC343756 | KC343998 |
| *Diaporthe cucurbitae* | CBS 136.25 | KC343031 | KC343757 | KC343999 |
| *Diaporthe arecae* | CBS 161.64 | KC343032 | KC343758 | KC344000 |
| *Diaporthe arecae* | CBS 535.75 | KC343033 | KC343759 | KC344001 |
| *Diaporthe arengae* | CBS 114979 | KC343034 | KC343760 | KC344002 |
| *Diaporthe aspalathi* | CBS 117168 | KC343035 | KC343761 | KC344003 |
| *Diaporthe aspalathi* | CBS 117169 | KC343036 | KC343762 | KC344004 |
| *Diaporthe aspalathi* | CBS 117500 | KC343037 | KC343763 | KC344005 |
| *Diaporthe australafricana* | CBS 111886 | KC343038 | KC343764 | KC344006 |
| *Diaporthe australafricana* | CBS 113487 | KC343039 | KC343765 | KC344007 |
| *Diaporthe batatas* | CBS 122.21 | KC343040 | KC343766 | KC344008 |
| *Diaporthe beckhausii* | CBS 138.27 | KC343041 | KC343767 | KC344009 |
| *Diaporthe brasiliensis* | CBS 133183 | KC343042 | KC343768 | KC344010 |
| *Diaporthe brasiliensis* | LGMF926 | KC343043 | KC343769 | KC344011 |
| *Diaporthe carpini* | CBS 114437 | KC343044 | KC343770 | KC344012 |
| *Diaporthe caulivora* | CBS 127268 | KC343045 | KC343771 | KC344013 |
| *Diaporthe caulivora* | CBS 178.55 | KC343046 | KC343772 | KC344014 |
| *Diaporthe celastrina* | CBS 139.27 | KC343047 | KC343773 | KC344015 |
| *Diaporthe chamaeropis* | CBS 454.81 | KC343048 | KC343774 | KC344016 |
| *Diaporthe chamaeropis* | CBS 753.7 | KC343049 | KC343775 | KC344017 |
| *Diaporthe cinerascens* | CBS 719.96 | KC343050 | KC343776 | KC344018 |
| *Diaporthe citri* | CBS 199.39 | KC343051 | KC343777 | KC344019 |
| *Diaporthe citri* | CBS 230.52 | KC343052 | KC343778 | KC344020 |
| *Diaporthe citri* | LGMF946 | KC343053 | KC343779 | KC344021 |
| *Diaporthe convolvuli* | CBS 124654 | KC343054 | KC343780 | KC344022 |
| *Diaporthe crataegi* | CBS 114435 | KC343055 | KC343781 | KC344023 |
| *Diaporthe crotalariae* | CBS 162.33 | KC343056 | KC343782 | KC344024 |
| *Diaporthe cuppatea* | CBS 117499 | KC343057 | KC343783 | KC344025 |
| *Diaporthe cynaroidis* | CBS 122676 | KC343058 | KC343784 | KC344026 |
| *Diaporthe decedens* | CBS 109772 | KC343059 | KC343785 | KC344027 |
| *Diaporthe decedens* | CBS 114281 | KC343060 | KC343786 | KC344028 |
| *Diaporthe detrusa* | CBS 109770 | KC343061 | KC343787 | KC344029 |
| *Diaporthe detrusa* | CBS 114652 | KC343062 | KC343788 | KC344030 |
| *Diaporthe detrusa* | CBS 140.27 | KC343063 | KC343789 | KC344031 |
| *Diaporthe elaeagni* | CBS 504.72 | KC343064 | KC343790 | KC344032 |
| *Diaporthe endophytica* | CBS 133811 | KC343065 | KC343791 | KC344033 |
| *Diaporthe endophytica* | LGMF911 | KC343066 | KC343792 | KC344034 |
| *Diaporthe endophytica* | LGMF919 | KC343067 | KC343793 | KC344035 |
| *Diaporthe endophytica* | LGMF928 | KC343068 | KC343794 | KC344036 |
| *Diaporthe endophytica* | LGMF934 | KC343069 | KC343795 | KC344037 |
| *Diaporthe endophytica* | LGMF935 | KC343070 | KC343796 | KC344038 |
| *Diaporthe endophytica* | LGMF937 | KC343071 | KC343797 | KC344039 |
| *Diaporthe endophytica* | LGMF948 | KC343072 | KC343798 | KC344040 |
| *Diaporthe eres* | CBS 101742 | KC343073 | KC343799 | KC344041 |
| *Diaporthe eres* | CBS 102.81 | KC343074 | KC343800 | KC344042 |
| *Diaporthe eres* | CBS 109767 | KC343075 | KC343801 | KC344043 |
| *Diaporthe eres* | CBS 110.85 | KC343076 | KC343802 | KC344044 |
| *Diaporthe eres* | CBS 122.82 | KC343077 | KC343803 | KC344045 |
| *Diaporthe eres* | CBS 129168 | KC343078 | KC343804 | KC344046 |
| *Diaporthe eres* | CBS 186.37 | KC343079 | KC343805 | KC344047 |
| *Diaporthe eres* | CBS 250.38 | KC343080 | KC343806 | KC344048 |
| *Diaporthe eres* | CBS 267.32 | KC343081 | KC343807 | KC344049 |
| *Diaporthe eres* | CBS 267.55 | KC343082 | KC343808 | KC344050 |
| *Diaporthe eres* | CBS 283.85 | KC343083 | KC343809 | KC344051 |
| *Diaporthe eres* | CBS 287.74 | KC343084 | KC343810 | KC344052 |
| *Diaporthe eres* | CBS 297.77 | KC343085 | KC343811 | KC344053 |
| *Diaporthe eres* | CBS 365.97 | KC343086 | KC343812 | KC344054 |
| *Diaporthe eres* | CBS 370.67 | KC343087 | KC343813 | KC344055 |
| *Diaporthe eres* | CBS 375.61 | KC343088 | KC343814 | KC344056 |
| *Diaporthe eres* | CBS 422.5 | KC343089 | KC343815 | KC344057 |
| *Diaporthe eres* | CBS 439.82 | KC343090 | KC343816 | KC344058 |
| *Diaporthe eres* | CBS 445.62 | KC343091 | KC343817 | KC344059 |
| *Diaporthe eres* | CBS 485.96 | KC343092 | KC343818 | KC344060 |
| *Diaporthe eres* | CBS 528.83 | KC343093 | KC343819 | KC344061 |
| *Diaporthe eres* | CBS 688.97 | KC343094 | KC343820 | KC344062 |
| *Diaporthe eres* | CBS 694.94 | KC343095 | KC343821 | KC344063 |
| *Diaporthe eres* | CBS 791.68 | KC343096 | KC343822 | KC344064 |
| *Diaporthe eres* | CBS 841.84 | KC343097 | KC343823 | KC344065 |
| *Diaporthe eres*^2^ | PC 17-288A | MH050348 |  |  |
| *Diaporthe eugeniae* | CBS 444.82 | KC343098 | KC343824 | KC344066 |
| *Diaporthe fibrosa* | CBS 109751 | KC343099 | KC343825 | KC344067 |
| *Diaporthe fibrosa* | CBS 113830 | KC343100 | KC343826 | KC344068 |
| *Diaporthe foeniculacea* | CBS 111553 | KC343101 | KC343827 | KC344069 |
| *Diaporthe foeniculacea* | CBS 111554 | KC343102 | KC343828 | KC344070 |
| *Diaporthe foeniculacea* | CBS 116957 | KC343103 | KC343829 | KC344071 |
| *Diaporthe foeniculacea* | CBS 123208 | KC343104 | KC343830 | KC344072 |
| *Diaporthe foeniculacea* | CBS 123209 | KC343105 | KC343831 | KC344073 |
| *Diaporthe foeniculacea* | CBS 171.78 | KC343106 | KC343832 | KC344074 |
| *Diaporthe foeniculacea* | CBS 187.27 | KC343107 | KC343833 | KC344075 |
| *Diaporthe foeniculacea* | CBS 287.56 | KC343108 | KC343834 | KC344076 |
| *Diaporthe foeniculacea* | CBS 357.69 | KC343109 | KC343835 | KC344077 |
| *Diaporthe foeniculacea* | CBS 400.48 | KC343110 | KC343836 | KC344078 |
| *Diaporthe foeniculacea* | CBS 603.88 | KC343111 | KC343837 | KC344079 |
| *Diaporthe ganjae* | CBS 180.91 | KC343112 | KC343838 | KC344080 |
| *Diaporthe gardeniae* | CBS 288.56 | KC343113 | KC343839 | KC344081 |
| *Diaporthe helianthi* | CBS 344.94 | KC343114 | KC343840 | KC344082 |
| *Diaporthe helianthi* | CBS 592.81 | KC343115 | KC343841 | KC344083 |
| *Diaporthe* cf. *heveae* 1 RG 201*3* | CBS 852.97 | KC343116 | KC343842 | KC344084 |
| *Diaporthe* cf*. heveae* 2 RG 2013 | CBS 681.84 | KC343117 | KC343843 | KC344085 |
| *Diaporthe hickoriae* | CBS 145.26 | KC343118 | KC343844 | KC344086 |
| *Diaporthe hongkongensis* | CBS 115448 | KC343119 | KC343845 | KC344087 |
| *Diaporthe hordei* | CBS 481.92 | KC343120 | KC343846 | KC344088 |
| *Diaporthe impulsa* | CBS 114434 | KC343121 | KC343847 | KC344089 |
| *Diaporthe impulsa* | CBS 141.27 | KC343122 | KC343848 | KC344090 |
| *Diaporthe inconspicua* | CBS 133813 | KC343123 | KC343849 | KC344091 |
| *Diaporthe inconspicua* | LGMF922 | KC343124 | KC343850 | KC344092 |
| *Diaporthe inconspicua* | LGMF931 | KC343125 | KC343851 | KC344093 |
| *Diaporthe infecunda* | CBS 133812 | KC343126 | KC343852 | KC344094 |
| *Diaporthe infecunda* | LGMF908 | KC343127 | KC343853 | KC344095 |
| *Diaporthe infecunda* | LGMF912 | KC343128 | KC343854 | KC344096 |
| *Diaporthe infecunda* | LGMF917 | KC343129 | KC343855 | KC344097 |
| *Diaporthe infecunda* | LGMF918 | KC343130 | KC343856 | KC344098 |
| *Diaporthe infecunda* | LGMF920 | KC343131 | KC343857 | KC344099 |
| *Diaporthe infecunda* | LGMF933 | KC343132 | KC343858 | KC344100 |
| *Diaporthe infecunda* | LGMF940 | KC343133 | KC343859 | KC344101 |
| *Diaporthe bicincta* | CBS 121004 | KC343134 | KC343860 | KC344102 |
| *Diaporthe longispora* | CBS 194.36 | KC343135 | KC343861 | KC344103 |
| *Diaporthe lusitanicae* | CBS 123212 | KC343136 | KC343862 | KC344104 |
| *Diaporthe lusitanicae* | CBS 123213 | KC343137 | KC343863 | KC344105 |
| *Diaporthe manihotia* | CBS 505.76 | KC343138 | KC343864 | KC344106 |
| *Diaporthe mayteni* | CBS 133185 | KC343139 | KC343865 | KC344107 |
| *Diaporthe megalospora* | CBS 143.27 | KC343140 | KC343866 | KC344108 |
| *Diaporthe melonis* | CBS 435.87 | KC343141 | KC343867 | KC344109 |
| *Diaporthe melonis* | CBS 507.78 | KC343142 | KC343868 | KC344110 |
| *Diaporthe musigena* | CBS 129519 | KC343143 | KC343869 | KC344111 |
| *Diaporthe neilliae* | CBS 144.27 | KC343144 | KC343870 | KC344112 |
| *Diaporthe neoarctii* | CBS 109490 | KC343145 | KC343871 | KC344113 |
| *Diaporthe* cf*. nobilis* RG 2013 | CBS 113470 | KC343146 | KC343872 | KC344114 |
| *Diaporthe* cf*. nobilis* RG 2013 | CBS 116953 | KC343147 | KC343873 | KC344115 |
| *Diaporthe* cf*. nobilis* RG 2013 | CBS 116954 | KC343148 | KC343874 | KC344116 |
| *Diaporthe* cf*. nobilis* RG 2013 | CBS 124030 | KC343149 | KC343875 | KC344117 |
| *Diaporthe* cf*. nobilis* RG 2013 | CBS 129167 | KC343150 | KC343876 | KC344118 |
| *Diaporthe* cf*. nobilis* RG 2013 | CBS 200.39 | KC343151 | KC343877 | KC344119 |
| *Diaporthe* cf*. nobilis* RG 2013 | CBS 338.89 | KC343152 | KC343878 | KC344120 |
| *Diaporthe* cf*. nobilis* RG 2013 | CBS 587.79 | KC343153 | KC343879 | KC344121 |
| *Diaporthe nomurai* | CBS 157.29 | KC343154 | KC343880 | KC344122 |
| *Diaporthe novem* | CBS 127269 | KC343155 | KC343881 | KC344123 |
| *Diaporthe novem* | CBS 127270 | KC343156 | KC343882 | KC344124 |
| *Diaporthe novem* | CBS 127271 | KC343157 | KC343883 | KC344125 |
| *Diaporthe novem* | CBS 354.71 | KC343158 | KC343884 | KC344126 |
| *Diaporthe novem* | LGMF943 | KC343159 | KC343885 | KC344127 |
| *Diaporthe oncostoma* | CBS 100454 | KC343160 | KC343886 | KC344128 |
| *Diaporthe oncostoma* | CBS 109741 | KC343161 | KC343887 | KC344129 |
| *Diaporthe oncostoma* | CBS 589.78 | KC343162 | KC343888 | KC344130 |
| *Diaporthe oncostoma* | CBS 809.85 | KC343163 | KC343889 | KC344131 |
| *Diaporthe oxe* | CBS 133186 | KC343164 | KC343890 | KC344132 |
| *Diaporthe oxe* | CBS 133187 | KC343165 | KC343891 | KC344133 |
| *Diaporthe oxe* | LGMF915 | KC343166 | KC343892 | KC344134 |
| *Diaporthe oxe* | LGMF939 | KC343167 | KC343893 | KC344135 |
| *Diaporthe oxe* | LGMF945 | KC343168 | KC343894 | KC344136 |
| *Diaporthe padi var. padi* | CBS 114200 | KC343169 | KC343895 | KC344137 |
| *Diaporthe padi var. padi* | CBS 114649 | KC343170 | KC343896 | KC344138 |
| *Diaporthe paranensis* | CBS 133184 | KC343171 | KC343897 | KC344139 |
| *Diaporthe perjuncta* | CBS 109745 | KC343172 | KC343898 | KC344140 |
| *Diaporthe perseae* | CBS 151.73 | KC343173 | KC343899 | KC344141 |
| *Diaporthe phaseolorum* | CBS 113425 | KC343174 | KC343900 | KC344142 |
| *Diaporthe phaseolorum* | CBS 116019 | KC343175 | KC343901 | KC344143 |
| *Diaporthe phaseolorum* | CBS 116020 | KC343176 | KC343902 | KC344144 |
| *Diaporthe phaseolorum* | CBS 127465 | KC343177 | KC343903 | KC344145 |
| *Diaporthe phaseolorum* | CBS 257.8 | KC343178 | KC343904 | KC344146 |
| *Diaporthe phaseolorum* | LGMF927 | KC343179 | KC343905 | KC344147 |
| *Diaporthe phaseolorum* | LGMF941 | KC343180 | KC343906 | KC344148 |
| *Diaporthe pseudomangiferae* | CBS 101339 | KC343181 | KC343907 | KC344149 |
| *Diaporthe pseudomangiferae* | CBS 388.89 | KC343182 | KC343908 | KC344150 |
| *Diaporthe pseudophoenicicola* | CBS 176.77 | KC343183 | KC343909 | KC344151 |
| *Diaporthe pseudophoenicicola* | CBS 462.69 | KC343184 | KC343910 | KC344152 |
| *Diaporthe pustulata* | CBS 109742 | KC343185 | KC343911 | KC344153 |
| *Diaporthe pustulata* | CBS 109760 | KC343186 | KC343912 | KC344154 |
| *Diaporthe pustulata* | CBS 109784 | KC343187 | KC343913 | KC344155 |
| *Diaporthe raonikayaporum* | CBS 133182 | KC343188 | KC343914 | KC344156 |
| *Diaporthe rhoina* | CBS 146.27 | KC343189 | KC343915 | KC344157 |
| *Diaporthe saccarata* | CBS 116311 | KC343190 | KC343916 | KC344158 |
| *Diaporthe schini* | CBS 133181 | KC343191 | KC343917 | KC344159 |
| *Diaporthe schini* | LGMF910 | KC343192 | KC343918 | KC344160 |
| *Diaporthe sclerotioides* | CBS 296.67 | KC343193 | KC343919 | KC344161 |
| *Diaporthe sclerotioides* | CBS 710.76 | KC343194 | KC343920 | KC344162 |
| *Diaporthe scobina* | CBS 251.38 | KC343195 | KC343921 | KC344163 |
| *Diaporthe sojae* | CBS 100.87 | KC343196 | KC343922 | KC344164 |
| *Diaporthe sojae* | CBS 116017 | KC343197 | KC343923 | KC344165 |
| *Diaporthe sojae* | CBS 116023 | KC343198 | KC343924 | KC344166 |
| *Diaporthe sojae* | CBS 127267 | KC343199 | KC343925 | KC344167 |
| *Diaporthe sojae* | CBS 180.55 | KC343200 | KC343926 | KC344168 |
| *Diaporthe sojae* | CBS 659.78 | KC343201 | KC343927 | KC344169 |
| *Diaporthe* sp. 1 RG 2013 | CBS 119639 | KC343202 | KC343928 | KC344170 |
| *Diaporthe* sp. 1 RG 2013 | LGMF947 | KC343203 | KC343929 | KC344171 |
| *Diaporthe* sp. 2 RG 2013 | LGMF932 | KC343204 | KC343930 | KC344172 |
| *Diaporthe* sp. 3 RG 2013 | CBS 287.29 | KC343205 | KC343931 | KC344173 |
| *Diaporthe* sp. 4 RG 2013 | LGMF944 | KC343206 | KC343932 | KC344174 |
| *Diaporthe* sp. 5 RG 2013 | CBS 125575 | KC343207 | KC343933 | KC344175 |
| *Diaporthe* sp. 6 RG 2013 | CBS 115584 | KC343208 | KC343934 | KC344176 |
| *Diaporthe* sp. 6 RG 2013 | CBS 115595 | KC343209 | KC343935 | KC344177 |
| *Diaporthe* sp. 7 RG 2013 | CBS 458.78 | KC343210 | KC343936 | KC344178 |
| *Diaporthe* sp. 8 RG 2013 | LGMF925 | KC343211 | KC343937 | KC344179 |
| *Diaporthe stictica* | CBS 370.54 | KC343212 | KC343938 | KC344180 |
| *Diaporthe subordinaria* | CBS 101711 | KC343213 | KC343939 | KC344181 |
| *Diaporthe subordinaria* | CBS 464.9 | KC343214 | KC343940 | KC344182 |
| *Diaporthe tecomae* | CBS 100547 | KC343215 | KC343941 | KC344183 |
| *Diaporthe terebinthifolii* | CBS 133180 | KC343216 | KC343942 | KC344184 |
| *Diaporthe terebinthifolii* | LGMF907 | KC343217 | KC343943 | KC344185 |
| *Diaporthe terebinthifolii* | LGMF909 | KC343218 | KC343944 | KC344186 |
| *Diaporthe terebinthifolii* | LGMF913 | KC343219 | KC343945 | KC344187 |
| *Diaporthe toxica* | CBS 534.93 | KC343220 | KC343946 | KC344188 |
| *Diaporthe toxica* | CBS 535.93 | KC343221 | KC343947 | KC344189 |
| *Diaporthe toxica* | CBS 546.93 | KC343222 | KC343948 | KC344190 |
| *Diaporthe vaccinii* | CBS 118571 | KC343223 | KC343949 | KC344191 |
| *Diaporthe vaccinii* | CBS 122112 | KC343224 | KC343950 | KC344192 |
| *Diaporthe vaccinii* | CBS 122114 | KC343225 | KC343951 | KC344193 |
| *Diaporthe vaccinii* | CBS 122115 | KC343226 | KC343952 | KC344194 |
| *Diaporthe vaccinii* | CBS 122116 | KC343227 | KC343953 | KC344195 |
| *Diaporthe vaccinii* | CBS 160.32 | KC343228 | KC343954 | KC344196 |
| *Diaporthe vexans* | CBS 127.14 | KC343229 | KC343955 | KC344197 |
| *Diaporthe rudis* | CBS 100170 | KC343230 | KC343956 | KC344198 |
| *Diaporthe rudis* | CBS 106.95 | KC343231 | KC343957 | KC344199 |
| *Diaporthe rudis* | CBS 109492 | KC343232 | KC343958 | KC344200 |
| *Diaporthe rudis* | CBS 109768 | KC343233 | KC343959 | KC344201 |
| *Diaporthe rudis* | CBS 113201 | KC343234 | KC343960 | KC344202 |
| *Diaporthe rudis* | CBS 114011 | KC343235 | KC343961 | KC344203 |
| *Diaporthe rudis* | CBS 114436 | KC343236 | KC343962 | KC344204 |
| *Diaporthe rudis* | CBS 266.85 | KC343237 | KC343963 | KC344205 |
| *Diaporthe rudis* | CBS 312.91 | KC343238 | KC343964 | KC344206 |
| *Diaporthe rudis* | CBS 446.62 | KC343239 | KC343965 | KC344207 |
| *Diaporthe rudis* | CBS 449.82 | KC343240 | KC343966 | KC344208 |
| *Diaporthe rudis* | CBS 502.85 | KC343241 | KC343967 | KC344209 |
| *Diaporthe rudis* | CBS 759.95 | KC343242 | KC343968 | KC344210 |
| *Diaporthe rudis* | CBS 794.96 | KC343243 | KC343969 | KC344211 |
| *Diaporthe woodii* | CBS 558.93 | KC343244 | KC343970 | KC344212 |
| *Diaporthe woolworthii* | CBS 148.27 | KC343245 | KC343971 | KC344213 |
